# Recommendations to enable drug development for inherited neuropathies: Charcot-Marie-Tooth and Giant Axonal Neuropathy

**DOI:** 10.12688/f1000research.3751.2

**Published:** 2014-04-22

**Authors:** Lori Sames, Allison Moore, Renee Arnold, Sean Ekins

**Affiliations:** 1 Hannah's Hope Fund, Rexford, NY, 12148, USA; 2 BioGAN Therapeutics, Rexford, NY, 12148, USA; 3 Hereditary Neuropathy Foundation, New York, NY, 10016, USA; 4 Arnold Consultancy & Technology LLC, New York, NY, 10023, USA; 5 Master of Public Health Program, Mount Sinai School of Medicine, New York, NY, 10029, USA; 6 Collaborations in Chemistry, Fuquay Varina, NC27526, USA; 7 Department of Pharmaceutical Sciences, University of Maryland, Baltimore, MD, 21201, USA; 8 Department of Pharmacology, Rutgers-Robert Wood Johnson Medical School, Piscataway, NJ, 08854, USA; 9 Division of Chemical Biology and Medicinal Chemistry, UNC Eshelman School of Pharmacy, University of North Carolina at Chapel Hill, Chapel Hill, NC, 27599-7355, USA

## Abstract

Approximately 1 in 2500 Americans suffer from Charcot-Marie-Tooth (CMT) disease. The underlying disease mechanisms are unique in most forms of CMT, with many point mutations on various genes causing a toxic accumulation of misfolded proteins. Symptoms of the disease often present within the first two decades of life, with CMT1A patients having reduced compound muscle and sensory action potentials, slow nerve conduction velocities, sensory loss, progressive distal weakness, foot and hand deformities, decreased reflexes, bilateral foot drop and about 5% become wheelchair bound. In contrast, the ultra-rare disease Giant Axonal Neuropathy (GAN) is frequently described as a recessively inherited condition that results in progressive nerve death. GAN usually appears in early childhood and progresses slowly as neuronal injury becomes more severe and leads to death in the second or third decade. There are currently no treatments for any of the forms of CMTs or GAN. We suggest that further clinical studies should analyse electrical impedance myography as an outcome measure for CMT. Further, additional quality of life (QoL) assessments for these CMTs are required, and we need to identify GAN biomarkers as well as develop new genetic testing panels for both diseases. We propose that using the Global Registry of Inherited Neuropathy (GRIN) could be useful for many of these studies. Patient advocacy groups and professional organizations (such as the Hereditary Neuropathy Foundation (HNF), Hannah's Hope Fund (HHF), The Neuropathy Association (TNA) and the American Association of Neuromuscular and Electrodiagnostic Medicine (AANEM) can play a central role in educating clinicians and patients. Undertaking these studies will assist in the correct diagnosis of disease recruiting patients for clinical studies, and will ultimately improve the endpoints for clinical trials. By addressing obstacles that prevent industry investment in various forms of inherited neuropathies, we can envision treatment options for these rare diseases in the near future.

## Introduction

There are currently no approved treatments for any of the forms of inherited neuropathy (IN), which are each considered rare diseases. Charcot-Marie-Tooth (CMT) Disease 1A is the most common form of IN. The underlying disease mechanisms are unique in the different forms of CMT ( *e.g.* CMT1A, CMT2, *etc.*) ^1^. To date, at least 51 causal genes have been implicated in CMT ^2^. CMT Type 1 diseases are demyelinating and Type 2s are axonal neuropathies ^3^. Based on the prevalence of all CMTs being 1:2500 and the recent census figures of over 300 million people in the United States of America (USA), it is likely that more than 75,000 Americans have CMT1A, which is caused by the peripheral myelin protein 22 (PMP22) duplication gene phenotype, which represents 68.7% of all CMT Type 1s in the USA ^4^. Symptoms of the disease often present in the first two decades of life ^5^, with patients having reduced compound muscle and sensory action potentials, slow nerve conduction velocities, sensory loss, progressive distal weakness, foot and hand deformities, decreased reflexes, bilateral foot drop and about 5% becoming wheelchair bound. Of the axonopathies, CMT2A is the most common form. CMT2A is primarily caused by mutations in the gene encoding mitofusin ( *MFN2*) ^6^. Mutations or rearrangements in *PMP22*, myelin protein zero (MPZ) and gap junction *β*1-protein (GJB1) or *MFN2* account for 90% of molecular diagnoses ^7^. CMT2A has been described to severely affect infants and children, with most affected individuals non-ambulatory by age 20, and having profound proprioception loss in addition to weakness ^6^.

Other than braces or other orthopedic devices, there are no effective therapies for any of the various forms of CMTs ^8^. Unfortunately, CMT1A is also a textbook case of how ground-breaking fundamental discoveries by academic scientists are not being rapidly translated into therapeutics quickly enough. For example, the causal gene duplication defect for the most common form of CMT (PMP22) was identified in 1991 ^9, 10^ and confirmation that the resulting elevated gene dosage is responsible for the pathology of the disease was established in 1993, yet the first high throughput screen (HTS) to search for a small molecule therapeutic was not published until 2012 ^11^. This represents a 21-year gap in translation from gene to screen. The tide is now slowly changing and a small pipeline of molecules currently exists for CMT1A. We are aware of at least two companies with preclinical and clinical compounds. Recently, a press release from Addex Therapeutics reported that the preclinical compound Addex ADX71441 reduces PMP22 expression in a dose-dependent manner comparable to baclofen in a pre-clinical transgenic mouse model of CMT1A disease. This drug is a new molecular entity that will require extensive preclinical testing, thus it is many years (possibly a decade) from US Food and Drug Administration (FDA) approval. Pharnext is a French pharmaceutical company which has just completed a Phase II trial (manuscript in preparation) for a combination therapy (baclofen, naltrexone and sorbitol) for CMT1A ^12, 13^. All these components are already FDA-approved, so if the Phase III clinical trial (proposed for 2014, Dr. Daniel Cohen, Pharnext personal communication) is successful, this could be the first treatment to market for any form of IN. In addition, the coming five years will likely be an explosive time in CMT2A research as the long-awaited, very difficult to engineer, CMT2A knock out mouse model is being characterized by Dr. David Pleasure (University of California, Davis). Preliminary data reveal that this model will be useful for better understanding the mechanisms behind the disease (molecular targets) — and will represent a putative model for testing the efficacy and safety of investigational therapeutics.

Recent clinical studies of other agents targeted for the treatment of CMT1A have not been successful, including the use of high-dose ascorbic acid, which exemplified the need for better therapeutic options ^14^. Further, a 5-year longitudinal study also using ascorbic acid in subjects with CMT1A, failed to identify a physiologically significant biomarker ^15^ in patients *vs* controls. Indeed, “ *at the end of the study, 30 patients (68%) and 2 controls (8%) stated that their physical condition had deteriorated during the 5-year study period*” ^15^. Verhamme *et al.* hypothesize that early weakness, summarized as *pes cavus*, lack of reserves, *etc.,* contribute to increased disability in patients, as they aged at the same rate as controls (same decrease in muscle strength and compound Muscle Action Potential [CMAP]) ^15^. The Inherited Neuropathy Consortium Rare Diseases Clinical Research Network (RDCRN) website (as of October 30 ^th^ 2013) lists the following six studies: 6601: Natural History Evaluation of Charcot Marie Tooth Disease; 6602: Genetics of CMT- Modifiers of CMT1A, New Causes of CMT2; 6603: Development of CMT Peds Scale for Children with CMT; 6606: An Analysis of the Symptomatic Domains Most Relevant to Charcot Marie Tooth Neuropathy (CMT) Patients; 6608: Survey of Current Management of Orthopedic Complications in Charcot Marie Tooth Disease Patients; 6604: Development and Validation of a Disability Severity Index for Charcot Marie Tooth Disease. All but studies 6604 and 6606 are listed as still recruiting. These clinical studies are also listed on clinicaltrials.gov with “unknown” status, indicating further the sub optimal progression of research studies in these diseases to date.

In contrast to CMT, which can vary in severity and is relatively prevalent, GAN is the most severe form of IN. GAN is an “ultra-rare” disease with tens of patients globally (with many likely to be undiagnosed ^16^), but neurologists suspect that some CMT Type 2 patients whose causal gene remains unknown may actually have GAN ^17^ greatly expanding the patient population. GAN generally appears in early childhood and progresses slowly as the neuronal injury becomes more severe. GAN is a single-gene, autosomal recessive disorder that is fatal by the late teens or early 20s. The *GAN* gene encodes the protein gigaxonin ^18^. Gigaxonin is an intracellular protein needed for long-term nerve survival. It is a predicted E3 ligase adaptor protein believed to flag substrates for ubiquination on the proteasome ^19^. Recent studies also suggest that disturbed cytoskeletal regulation, likely involving the proteasome degradation pathway ^20^, is responsible for the formation of aggregates of some type 3 and type 4 intermediate filament proteins, which is a morphological characteristic of this disease, and many others.

Based on histopathological analysis of large axonal swellings, “giant axons” are filled with neurofilament (NF) triplet proteins (neurofilament light chain [NF-L], neurofilament medium chain [NF-M] and neurofilament heavy chain [NF-H]) in both the central and peripheral nervous systems of GAN patients ^21^. The knockout GAN -/- mouse has a similar histological phenotype, with increased levels of NF proteins, α-internexin, peripherin, as well as vimentin, with mRNA transcription levels remaining normal. Additionally, histological analysis of brain sections from the GAN -/- mouse has revealed accumulations of NF-H and of α-internexin in the cortex (negative for other NF subunits) ^21^.

Using three cellular models, Mahammad *et al.* ^20^ showed that restoring functional gigaxonin in cultured cells had a direct impact on vimentin, peripherin and NF-L. Using GAN patient fibroblasts, they were able to clear the vimentin aggregates by expressing wild type (WT) gigaxonin. Using a rat PC12 cell line stably expressing WT gigaxonin, they were also able to show the loss of peripherin in these cells. Lastly, using a human neuroblastoma cell line, SH-SY-5Y that stably expresses WT gigaxonin, they revealed clearance of NF-L protein by both immunoblotting and immunofluorescence. Since their manuscript ^20^ was accepted for publication, the Goldman and Opal laboratories have obtained evidence that gigaxonin also regulates the turnover of glial fibrillary acidic protein (GFAP) in astrocytes (Dr. Robert Goldman, Northwestern University Feinberg School of Medicine, personal communication).

Likewise, Mussche *et al.* were able to demonstrate proof-of-concept of GAN gene delivery *in vitro* and *in vivo* by clearing IF aggregates. They showed that the restoration of WT gigaxonin with adeno-associated virus vector serotype 2 (AAV2) clears vimentin IF aggregates in the fibroblasts of GAN patients. Using an AAV9 viral vector, (the same vector being used in the *GAN* gene delivery clinical trials that HHF is sponsoring), GAN knockout mice received an intracisternal injection of an AAV9/GAN vector that allowed the delivery of the GAN gene to the brainstem and spinal cord. The authors observed that “the treated mice showed a nearly complete clearance of peripherin IF accumulations in the brainstem and spinal cord at 3 weeks post-injection” ^22^. These studies demonstrated that gigaxonin gene transfer can reverse the cellular IF aggregate pathology associated with GAN. Based on this and other published studies (described earlier), it is fair to speculate, that vimentin, peripherin, NF-L, NF-M, NF-H, α-internexin and GFAP could be used as putative surrogate biomarkers of disease severity.

As GAN progresses (through effects on the CNS), patients typically become quadriplegics, dependent on a feeding tube and ventilator, and usually die in the second or third decade. The majority of GAN patients that have been identified to date have also had extremely kinky hair. However, there are two confirmed GAN cases with straight hair, further suggesting CMT as a differential diagnosis for GAN ^23^. Some pathological factors in GAN, like neurofilament light chain (NF-L) and peripherin accumulation, are also hallmarks of many other diseases ^24^ including Alexander disease (A×D), amyotrophic lateral sclerosis (ALS), Alzheimer’s disease, Parkinson’s disease, dementia with Lewy bodies, neuronal IF inclusion disease (NIFID), diabetic neuropathy, spinal muscular atrophy (SMA), and some forms of CMT (such as CMT 2E) ^20, 25^]. Thankfully, basic cellular biology research has caught up with clinical research for GAN. Patient cerebrospinal fluid (CSF) samples that have been collected over the past three years can finally be used in pilot studies now that putative surrogate biomarkers have finally been elucidated ^20^.

Over the past five years, HHF, through grassroots efforts, has funded the development and study of a novel GAN gene delivery investigational new biologic (INB) for the treatment of GAN. The gene delivery product is an AAV9 capsid with a self-complementary genome containing the WT-gigaxonin transgene (Dr. Steven Gray, UNC personal communication). This group has achieved broad distribution of the vector throughout the central nervous system (CNS) of both non-human primates and pigs with transduction of the transgene found even deep into the brain parenchyma ^22^. NINDS is sponsoring the Phase I trials that will take place at the National Institute of Health (NIH) Clinical Center in Bethesda, MD, USA. US Recombinant DNA Advisory Committee (RAC) approval was obtained during a public hearing in Washington DC that took place on June 12, 2013. Assuming FDA and (IRB) approvals, a Phase I trial is expected to begin in the second quarter of 2014.

With research reaching such advanced stages we propose that some efforts should be made to fill the various gaps in clinical studies, in order to translate therapeutics to patients more rapidly for the related diseases CMT1A, CMT2A and GAN. An important component will be to train clinicians, identify the diseases and provide information to hereditary neuropathy patients to help them manage their condition. In addition, there need to be continued efforts to bring new patients to the attention of researchers for clinical studies and energize pharmaceutical companies and investors to pursue treatments for hereditary neuropathies.

We propose that in order to translate these preclinical advances to the clinic and result in successful therapeutics there should be 1) alternative outcomes measures for CMT; 2) demonstration that potential treatments are having an effect on the QoL of patients; 3) genetic testing panels to facilitate identification of patients with CMT and GAN for future clinical trials; 4) validation of putative CSF biomarkers for GAN; 5) use of the GRIN database to capture patients for future clinical trials and 6) education of clinicians and patients as to therapeutic options for these diseases. Each of these topics is discussed further below.

### Alternative outcome measures: electrical impedance myography

The development of therapies for patients critically depends on the outcome measures used in clinical trials. There are limited efforts to find additional outcome measures for CMT, including *in vivo* confocal microscopy of Meissner corpuscles ^26^, Anterior Tibialis CMAP amplitude ^27^, CMT neuropathy score (second version) ^28^ and patient identified QoL ^29^. We now propose that a promising technique called electrical impedance myography (EIM) could be validated as an outcome measure for CMT1A and CMT2A disease progression, facilitating the execution of therapeutic CMT clinical trials in patients.

Skulpt, Inc. (previously Convergence Medical Devices, Inc.) has developed a handheld device specifically for performing EIM measurements in patients. EIM measures the impedance of skeletal muscle over a frequency range between 1 kHz and 10 MHz. The impedance is measured at each frequency by applying low-intensity electrical current *via* surface electrodes and measuring the resulting voltage signals using a second set of surface electrodes. EIM involves only minimal risk. The estimated testing duration for an individual subject is 15 to 30 minutes, depending on the number of muscles tested. The data obtained with this handheld EIM device have shown marked alterations between healthy individuals and patients. Data from both human subjects and animal disease models, including amyotrophic lateral sclerosis (ALS), Spinal muscular atrophy (SMA), Duchenne muscular dystrophy (DMD) and neurogenic mouse models, show that EIM may be sensitive to a variety of pathological states ^30– 34^. We envisage that EIM will help quantify the severity of the disease affecting various muscle groups as well as measure changes in the progression of the disease over time, which are critical aspects to determine if a therapeutic has any value. A recent DMD EIM pilot study enrolled 61 boys with DMD and 31 healthy controls and validated whether EIM allowed detection of diseased muscle tissues *vs* control tissues ^30^. Further, EIM was helpful in quantifying the change in muscle quality over time (Dr. Eugene Williams, Dart Therapeutics, personal communication). This was a single day evaluation study that showed strong inter- and intra-rater reliability. It was also a cross sectional study, which used boys over a wide age range, and allowed to correlate EIM readings with age and measures of disease progression ^30^. While this is not technically showing changes over time (a true “longitudinal study”), it does provide similar information and presents some valuable preliminary data.

Thus, EIM represents a novel, cutting-edge technology that deserves to be evaluated as to its validity in CMT and other INs.

### Demonstration that potential treatments are having an effect on the quality of life of patients

Although CMT is typically a slowly progressive disease, it may have a profound negative impact on patients’ functional, emotional and social abilities, resulting in reduced QoL. A number of studies of the influence of CMT on QoL have been published in the past several years; however, they have been either in small numbers of patients, with non-validated instruments, and/or specific to non-US locations, which reduce their generalizability to US populations ^29, 35– 37^. These studies have employed generic QoL instruments, such as the EQ-5D ^35, 38^, SF-36 ^36, 37, 39^, the Child Health Questionnaire ^40, 41^, and qualitative interviews using a semi-structured approach and open-ended questions ^29^. Moreover, different evaluations have produced opposing results. For example, a study of 20 Italian patients with follow-up at two years found worsening strength of distal muscles of upper limbs and proximal muscles of lower limbs, sensory function and walking disability, but no worsening of QoL, as measured by the SF-36 ^36^. Due to the small number of patients, it was not possible to stratify according to disease severity, which may have confounded the results. The researchers hypothesized that this may have been due to adaptive mechanisms of the patients because of the slow progression of disease or lack of sensitivity of the QoL instrument to changes specific to CMT. All of these authors ^36^ called for the development of CMT1A disease-specific QoL instruments (adult and child) as there is currently no disease-specific QoL instrument for CMT. Disease-specific instruments are more responsive to small, but important, changes in health than are generic instruments ^42^. A disease-specific instrument for CMT1A would have enhanced validity (having it measure what was intended), reliability (the extent to which it discriminates between individuals in a population in a consistent manner), sensitivity to change (the ability to measure true change regardless of whether it is relevant or meaningful to the patient or clinician) and responsiveness (the ability to detect change that is important to the patient) ^43^.

Johnson and colleagues ^29^ recently completed a study using a comprehensive qualitative interview method to identify symptoms and themes that had the greatest impact on CMT1A-related QoL in 16 adult patients. The themes mentioned most frequently were difficulty with mobility and ambulation, specific-activity impairment and emotional distress, while the most frequently noted individual symptoms in this study included impaired walking, falls and tripping. It is likely given the small number of patients, these did not correlate with CMT Neuropathy Score (CMTNS) as an indication of disease severity.

Padue *et al.* recently described a survey of CMT patients and caregivers and their perspectives and perceptions of efficacy and needs ^44^. This cross sectional study used the Rehabilitation Access Questionnaire, the SF36 QoL questionnaire and the family APGAR. This observational survey of 123 patients enrolled through clinical and genetic testing, suggested that patients believe it is important to feel better after physical therapy. There was a discrepancy between the perception of benefit from rehabilitation for the patient *vs* the caregiver.

Barrett and Birdsall ^45^ also completed an online qualitative study of adults (N=82) that used a modified van Kaam phenomenological method to explore (until saturation) thoughts, perceptions and feelings, including physical, emotional, and social effects. In addition, the study explored their sense of Barrett’s power as *knowing* participation in change—involving their ability to make informed choices with the intention of freely involving themselves in creating desired changes. The extent of the impact varied with severity, attitude, coping methods, resources, available interventions and treatments. Lack of awareness in the medical community was a predominant theme, as well as extensive description of the physical, emotional, and social impacts. Regarding personal power, searching for ways to educate themselves regarding their disease, to access quality of care, to feel fulfilled despite the severity of symptoms and ways to improve QoL were themes that occupied awareness (A). Regarding choices (C), many patients reflected the ways they were coping with physical limitations and stress, choosing protective and beneficial actions, as well as choosing attitudes taking advantage of available resources, taking charge of health decisions, giving up destructive habits, finding hobbies, altruistic actions, bringing information to physicians, and making necessary career changes. Regarding freedom to act intentionally (F), the range was from most who felt no freedom to those feeling absolutely free in relation to CMT. This was most often related to available and adequate medical care by physicians who “cared”. Regarding involvement in creating change (I), being able to find the best and affordable medical care and research findings often allowed people to make major changes. Each of these four power dimensions (A, C, F, I) represent areas for different groups ( *e.g.* Hereditary Neuropathy Foundation [HNF] or others) to address when planning interventions to guide patients to on how they can be empowered to improve their QoL. In addition, 13 QoL quantitative questions were explored by Barrett and Birdsall ^45^ using descriptive statistics.

In summary, variable results have been found in terms of QoL domains affected, with some studies showing that social and emotional aspects were significantly affected by the disease ^37^, and others showing no long-term influence on social and emotional functioning, but instead demonstrating impacts on physical and mental health ^36^. Moreover, the impact of disease progression on QoL is unclear, as some researchers have found paradoxical outcomes of worsening disease with no or only mild deterioration in QoL ^29, 36^. This is further confounded by a research study in children with CMT1A, where physical signs and symptoms were found to be of significantly greater value than psychological aspect of QoL, as relayed by their parents ^40^. Since there is no clear delineation of disease progression symptoms, relationship to disease severity, longitudinal follow-up, determination of best methods of measuring QoL or disease-specific instrument, future studies should evaluate changes in impact of factors. These factors should include improved medical care, education through the Internet, advocacy support from CMT organizations and other dynamics, on patients’ quality of life over the past ten years. Also newer instruments, such as NIH’s Patient Reported Outcomes Measurement Information System (PROMIS ^®^) and Neuro-QOL, need to be used in comparison to more established tools to determine if any of these tools is/are responsive enough to capture the impact of these diseases on patients’ health-related QoL (HRQoL). Neuro-QOL is a new, standardized approach to measuring HRQoL across common neurologic diseases and has been validated in multiple neurological conditions, both in adult and pediatric patients ^46^. PROMIS ^®^ is a tool defined as “ *a system of highly reliable, precise measures of patient–reported health status for physical, mental, and social well–being*” ^47^. Further psychometric testing within CMT and between CMT and GAN will help evaluate the generalizability of these new tools. Ultimately, there should be separate development and validation of CMT1A (disease-specific) QoL instruments for adults and children for use in future clinical trials.

### Develop genetic testing panels for CMT and GAN

INs are widely known as diverse in both their genetic causes and clinical phenotypes. INs are also very frequently underdiagnosed or misdiagnosed ^48^. In 2013 a large prospective study found that nearly half of the patients referred to specialist peripheral neuropathy clinics have an unrecognized inherited cause ^49^. We are aware that the sequencing company Athena/Quest Diagnostics has sequenced more than 100,000 CMT patients and claims to have the largest variant database in the world. Previous discussions have suggested that published prevalence data are outdated, and that CMT is much more prevalent than previously estimated (Dr. Stephen L. Vincent, Director of Business Development, Quest Diagnostics and Athena Diagnostics, personal communication). It is therefore important that updated prevalence data be communicated to illustrate the large and underserved patient population in order to motivate the pharmaceutical industry to develop treatments for CMT. There should therefore be coordinated efforts for the genetic diagnosis of this community with initial focus on CMT1A, CMT2A and GAN in order to have well-defined cohorts available for upcoming clinical studies.

To date, 2000 variants have been identified on the causal gene in cystic fibrosis and 18 causal variants have been identified (7). Perhaps CMT should be considered in the same light as the Cystic Fibrosis Foundation/Vertex Pharmaceuticals apparent view of cystic fibrosis, in which each genotype (e.g. mutations G551D, Delta F508 *etc*.) is treated as a different disease, requiring separate drugs for patients with different mutations. This ultimately represents an approach to individualized therapy. For INs, a long journey awaits as the field is working to elucidate causal genes in many cases, not to mention the many variants on a given gene that mandate a unique therapeutic approach. This is in stark contrast to the belief and understanding of most neurologists who are content in not investigating the genetic diagnosis of patients because no treatments currently exist. This paradigm must change in order to better understand the variability in the different forms of IN. The genotype must be identified and non-disease causing variants evaluated, as this can lead to the development of therapeutic targets. With the advancements in other personalized medicine approaches such as antisense oligonucleotides, we must endeavor to obtain a thorough understanding of the genetics behind the various forms of IN; otherwise it will be difficult to obtain effective therapies. In our view the field of IN is at least 20 years behind cystic fibrosis, largely because genetic testing has not been a priority. We have coined the phrase “diagnostic complacency” to describe this situation. Just because most forms of IN are not fatal and no treatments exist, it does not mean that CMT and idiopathic neuropathy patients do not deserve to be genetically diagnosed and have the opportunity to contribute to research by sharing their phenotype/genotype relationships.

Athena/Quest Diagnostics currently provides a variety of CMT next-generation sequencing options and plans on adding newly identified genes to these test panels in early 2015 (Dr. Stephen L. Vincent, Quest Diagnostics and Athena Diagnostics, personal communication). In addition, soon they may be able to provide disease-specific exome sequencing. This will allow them to test all known CMT genes in a single test in the same timeframe that it takes to run a single test today. We are not aware of other companies doing sequencing for CMTs and GAN specifically, therefore Athena’s/Quest’s CMT next-generation sequencing test offerings may provide a rapid result and cost savings for idiopathic CMT subjects in contrast to running multiple single or small test panels over an extended period of time.

The development of new CMT panels that include the GAN gene and other newly-identified CMT Type 2 genes that have a phenotype and electrophysiological findings similar to GAN will be important now that a Phase I trial is planned for GAN.

### GAN biomarker study

In order to assist in Phase I and Phase II trials of a first-in-human gene delivery vector delivered to the spinal cord there should be validated putative surrogate biomarkers of GAN disease progression. CSF has long been exploited as a relatively non-invasive way to sample the CNS ^50^. Subjects with GAN will likely be the first disease community in the world to receive a therapeutic gene delivered to the spinal cord, paving the way for other related neurological disorders such as spinal muscular atrophy (SMA) which, according to the National Organization for Rare Disorders (NORD), is the leading genetic cause of death in children.

As it has been reported that gigaxonin regulates GFAP in astrocytes ^51^, quantifying the levels of GFAP in body fluids may be useful to determine the severity and/or clinical progression of GAN. Normally, low levels of GFAP are seen in body fluids, and will thus require the development of very sensitive detection methods, such as sandwich ELISA assays ^52^. CSF GFAP levels have been shown to be elevated in a number of neurological conditions, reflecting a wide range of etiologies (reviewed in ^53^). In several situations GFAP is evaluated relative to other biomarkers linked to neuronal damage, such as neurofilaments ^54, 55^. Generally, the levels of GFAP are modest in the slowly progressive conditions such as dementia and multiple sclerosis (MS), and in contrast, higher but transient, in vascular accidents, trauma, and infection. The level of GFAP in CSF may have a prognostic value ^56– 58^ which is worth exploring. For instance, according to a study on patients with subarachnoid hemorrhage, six days after he event the GFAP level was approximately 8-fold higher in non-survivors compared to survivors ^58^. It has been described that GFAP levels in CSF seem to drop in response to treatment in patients with neuromyelitis optica given corticosteroid therapy ^59^.

Alexander disease (closely related to GAN) is caused by dominantly-acting gain of functional mutations in GFAP. These mutations lead to increased levels of expression, measured either by mRNA or protein. GFAP has been measured in the CSF from three Alexander disease patients ^60^, and was elevated in them all. Since mutations in GFAP are the root cause of Alexander disease, and increased levels of GFAP within astrocytes are thought to be central to the pathogenesis ^61^, GFAP measurements might prove especially valuable for assessing either severity or progression of this particular condition. For instance, the highest levels of GFAP in this study were found in a patient who, although intermediate in terms of age of onset, was rapidly deteriorating at the time of biopsy. Recent studies in the mouse model of Alexander disease also showed a positive correlation between the levels of protein present in brain parenchyma and the levels of protein in CSF, with an increased expression correlating with the severity of phenotype ^62^.

It is perhaps not surprising that Alexander disease and GAN show interesting similarities. The hallmark pathological feature in Alexander disease is the presence of stress protein aggregates (known as “Rosenthal fibers”) within the cytoplasm of astrocytes, along with variable degrees of leukodystrophy. Indeed, the presence of Rosenthal fibers has also been described in several patients with GAN ^63– 65^. It is intriguing to speculate therefore that loss of gigaxonin affects GFAP, causing accumulation and aggregation of this protein, in a manner similar to its effects on other IFs, and may even produce Alexander disease–like pathology. A recent authoritative review on the MRI features of leukodystrophies commented on the similarities between GAN and Alexander disease but was limited to focusing on lesions in the white matter ^66^. We could predict that GFAP levels will be increased in the CSF of GAN patients and will likely rise with the progression of the disease. We further hypothesize that GFAP levels will stabilize and potentially fall in response to treatment, thus serving as a useful biomarker for the GAN gene delivery clinical trial. It will be interesting to see if the abundance of GFAP, NF-H, NF-M, NF-L, vimentin, α-internexin and peripherin (all proteins implicated in GAN morphology) in CSF, changes over time and correlates with disease severity in the future trial. It is also intriguing to think that correlating NF-L abundance in CSF to disease progression in GAN may also translate to CMT2E, which is caused by dominant point mutations in NF-L. Most intriguing is the prospect that the markers for this devastating disorder in the pilot study could potentially be valuable surrogate endpoints demonstrating efficacy of the first-in-human AAV9 GAN gene delivery trials.

### Global Registry for Inherited Neuropathy (GRIN)

**Supplemental Figure 1.**
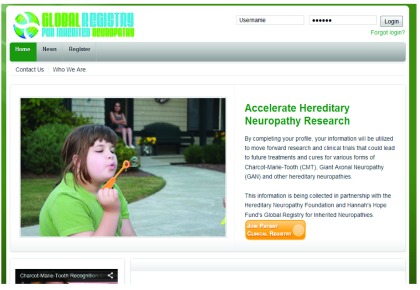
Website landing page for Global Registry for Inherited Neuropathy (GRIN).

**Supplemental Figure 2.**
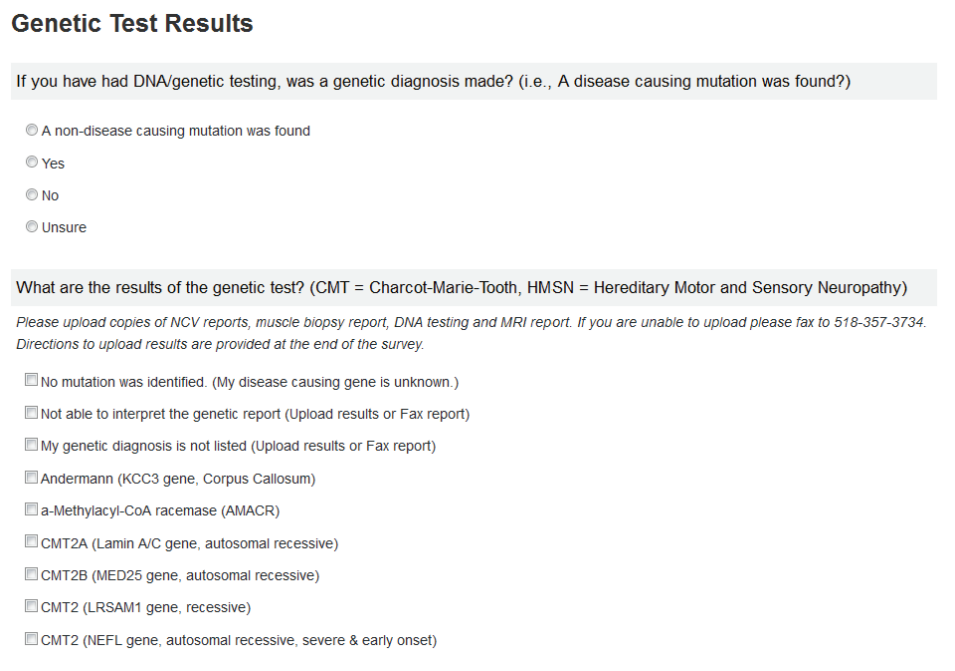
Genetic tests.

**Supplemental Figure 10.**
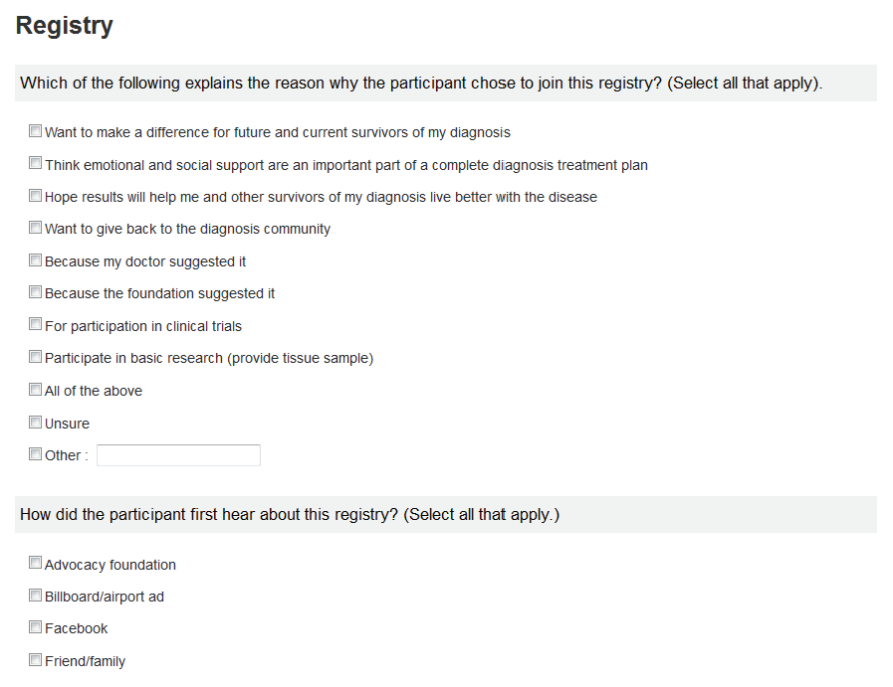
Registry.

A patient registry is a place to store detailed information about patients with a specific disease or syndrome. In general, collection, analysis and dissemination of data on disease progression and patient responses to long-term disease management strategies represents a valuable way to improve understanding of the disease and keep medical professionals up to date on the latest advances. The current Inherited Neuropathy Consortium (INC) has a very basic contact registry only for CMT1A, CMT1B, CMT2A, CMTX, CMT4, other known CMT peripheral neuropathy and other unknown CMT peripheral neuropathy. In contrast, the HNF & HHF launched the Global Registry for Inherited Neuropathies (GRIN, Supplemental Figure 1) – in 2013 with the primary objective of identifying patients with INs to gain a better understanding of disease phenotypes, which vary among all the different types and sub-types of CMT and related INs. This registry is far more extensive ( Supplemental Figure 2– Supplemental Figure 10) than anything else available for INs such as basic contact registries, as it covers all INs and is managed by Patient Crossroads, which is a professional organization that has developed registries for more than 250 diseases. These registries are used by participants in more than 75 countries. GRIN can be used to connect patients, healthcare providers, researchers, clinical investigators, regulators, policy-makers industry and beyond.

The GRIN collects data on patients who have either been clinically diagnosed by a doctor or genetically diagnosed with a form of IN ( Supplemental Figure 1). Establishing the registry addresses three critical needs. First, the scientists who study various forms of IN need accurate information to understand the specific forms of neuropathy; second, the scientists who are conducting preclinical studies may need tissue samples from registrants who have already agreed to be contacted by GRIN if samples are needed to advance research for their form of IN. Additionally, clinicians, pharmaceutical and biotechnology companies ready to begin trials would need the GRIN administrator to distribute and advertise the study and clinical-trials.gov study links. The Patient Crossroads platform on which the database is built provides robust query functionality and allows researchers to narrow in on specific phenotypes and genotypes. If a patient looks like a good match for research, the scientist will contact HNF. The foundation or its agent will then contact the IN family. Scientists will not contact the families directly and will only be given de-identified patient data.

Although there is no direct benefit to any patients participating in the registry, by collecting information on patients in the registry, scientists can:

- Study why individuals have different symptoms.
- Learn about phenotype/genotype relationships (how a particular mutation type can lead to different or unique symptoms).
- Learn how future treatments work or don’t work for a given patient population.
- Obtain tissue samples from registrants who agreed to be contacted for such purpose.
- Help medical professionals improve how they treat patients with IN, for example, pain management.
- Speed up research in INs by collecting information that scientists can use.
- Let patients (or their families) know when they may be eligible for clinical research studies (clinical trials).
- Let pharmaceutical companies know the number of patients with a particular form of IN who would be willing to consider volunteering for experimental trials.

This longitudinal database of patient-contributed data includes the ability for patients to create an account that allows them to login and update information, complete questionnaires or provide patient-reported outcome measures and track results, compare their responses to the de-identified responses of other registry participants, and upload medical records for use in identifying confirmed diagnosis of hereditary neuropathy patients. GRIN also provides the ability to monitor registration metrics, respond to registrant inquiries, add researcher-specific questions, and export de-identified data in CSV format that is widely accepted by statistical and scientific software.

GRIN currently collects data on meaningful characteristics such as *pes cavus*, absent-to-poor reflexes, toe walking, mobility devices on various levels of disability deficits, *etc*., management, and outcomes of disease progression with and without palliative treatments. Another critical component of the registry is to provide a better understanding of disease progression in the general population of both diagnosed and undiagnosed patients. Environmental factors and contraindicated drugs have been confirmed to exacerbate CMT and/or present onset of symptoms in asymptomatic patients ^67^. This obviously can alter disease progression and may be relevant to the natural history and clinical outcomes of treatments.

The registry has several objectives:

1. Offer additional data to enable the pharmaceutical industry to judge the risk – reward of the patient population for pursuing future clinical trials.
2. Obtain a better understanding of natural history from the perspective of the patient. Currently the patient, caregiver or parent of a minor, answers the questions, and when tests (needle electromyography, nerve conduction velocities or genetic tests) are available, patients and/or physicians upload the results to the registry or contact HNF directly.
3. Evaluate safety, efficacy, and influence of therapy on patients’ QoL, which is not currently a feature of any IN registry.
4. Record patient experience in a period prior to baseline as a control for future clinical trials.
5. Help to establish relationship of genotype – phenotype on disease progression.
6. Help to define data that are clinically meaningful.

GRIN includes a comprehensive series of questions covering many areas and samples of these are now provided for: diagnosis/assessments, genetic test results, general health, neurology, orthopedic, alternative/complimentary therapies, skin/hair, clinical trials, and research/bio-repository ( Supplemental Figure 1– Supplemental Figure 10). GRIN will help close the data gap to enable a more accurate representation of the patients’ experience. The registry contains well thought out questions and was beta tested by patients, caregivers and medical professionals. GRIN is also IRB-approved through the Chesapeake IRB. HNF’s diverse team of medical advisors, industry leaders, and the data used from focus groups conducted and analyzed through the National CMT Resource Center in 2010 helped to develop the purpose, objective, feasibility and the robust registry. GRIN is being marketed globally through aggressive pay-per-click campaigns (via a Google grant) to actively search out patients with CMT and other neuropathies. These marketing efforts ask them to join the registry and encourage them to be seen at one of the currently-funded RDCRN CMT Centers, Muscular Dystrophy Association Centers or by medical professionals trained to recognize, diagnose and treat CMT where patients are not economically able or are too severely affected to travel.

### Training and advocacy

If we are to develop new technologies and drugs for IN then we need to continually train clinicians to be aware of the state of the art knowledge of the diseases such as the growing list of known neurotoxins that must be avoided. At the same time we need to educate patients about their disease and what is in the research and clinical pipeline. The training of future physician-scientists is therefore critical to accelerating research in the hereditary neuropathies field. There is also the responsibility of foundations like HNF to educate the entire medical community to be aware of hereditary neuropathies. This would lead to faster and more accurate patient diagnosis that could have an impact on medical expenses and patient QoL.

Close affiliations with other patient advocacy organizations focused on INs is also important to educate all members of the community. As indicated throughout this document, key advocates include: HNF, HHF, The Neuropathy Association, Neuropathy Action Foundation, and NORD. To date HNF has actively supported the efforts of the RDCRN Inherited Neuropathies Consortium by including new studies for recruitment in monthly newsletters to over 7,000 members and aggressive social media outreach. Platforms used include Facebook, Twitter and the CMT Inspire Community. With well over 1,000 patients, the Inspire CMT community has dramatic branding and is a highly targeted and engaged population with valuable content for patients and clinicians. Inspire offers access to members for clinical studies that include market research and awareness campaigns where each member receives daily or weekly e-mails from Inspire — all aimed at getting the community to take action and move research forward. The mission of the Neuropathy Association is to bring help, hope, and healing to the estimated over 20 million people living with over 100 various forms of neuropathy. A significant segment of this population has an idiopathic neuropathy. Experts in the field have found that there is a high likelihood that many of these are actually unrecognized inherited neuropathies. The Neuropathy Association’s extensive network of patients, through their membership base (over 50,000), extensive online and social media networks (35,000–45,000 unique website visitors per month and 12,000 followers on Facebook) and the 15 designated Centers of Excellence at prominent academic and medical institutions across the USA, are critical to engaging clinicians – and their patients - to participate in future clinical trials. The Neuropathy Association and the HNF recently hosted a monthly Facebook Chat (July 2013), focusing on hereditary neuropathies, with the goal of giving participants a chance to hear from — and ask questions to — health experts in the field, the Association and Foundation staff, collaborating patient advocacy groups, as well as each other. These examples show that by actively engaging the advocacy groups and their networks through social media we can reach patients which, in previous years, would have been impossible without extensive media expenditures.

The Neuropathy Association has awarded grants for scientific research on hereditary neuropathies and the supported research projects have yielded significant results. For example, Landsverk *et al.* identified the gene causing a hereditary neuropathy (hereditary neuralgic amyotrophy [HNA]) that attacks the muscles responsible for controlling the movement of shoulders and arms ^68^. In addition, Florian Thomas (St. Louis University, St. Louis, USA) and his international team have uncovered three different genetic mutations specific to dominant intermediate CMT neuropathy type C, a form of CMT disease ^69^. Organizations like The American Association of Neuromuscular and Electrodiagnostic Medicine (AANEM) could be funded to support awarding of fellowships that focus on rare neuromuscular disease research. This could have a significant impact on training and education for the IN clinical community.

Additionally, a regular Hereditary Neuropathies Workshop could serve as a forum to promote awareness of IN, encourage patient recruitment for future clinical trials and GRIN, and provide a means for encouraging more participation by other researchers and companies in the field, as well as provide a learning platform to benefit new researchers; this would encourage the researchers’ development in this area and promote their interest in pursuing additional research projects in the field of hereditary neuropathies.

## Conclusion

In conclusion, we have recognized the following gaps in translation and obstacles to future clinical studies for IN *via* lack of: a validated QoL instrument that is sensitive enough to capture disease impact; validated biomarkers that correlate with severity of disease progression; a large, robust, clinical patient registry that accurately captures severity and range of symptoms, along with genotypes; physician education and awareness about IN; accurate genetic diagnoses; patient and physician education regarding the need for an early diagnosis; prevalence data; data regarding patient willingness to consider volunteering for experimental trials for various forms of IN. We have suggested some recommendations that could perhaps remedy this situation--positively impacting patients with CMT, GAN and other INs.

## Figures and Tables

**Supplemental Figure 3.**
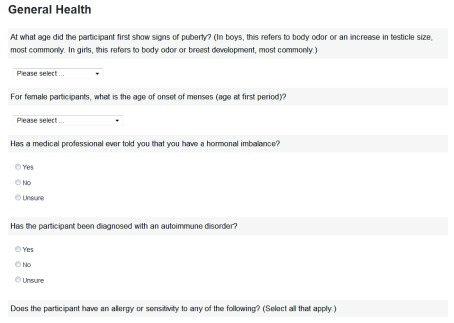
General health.

**Supplemental Figure 4.**
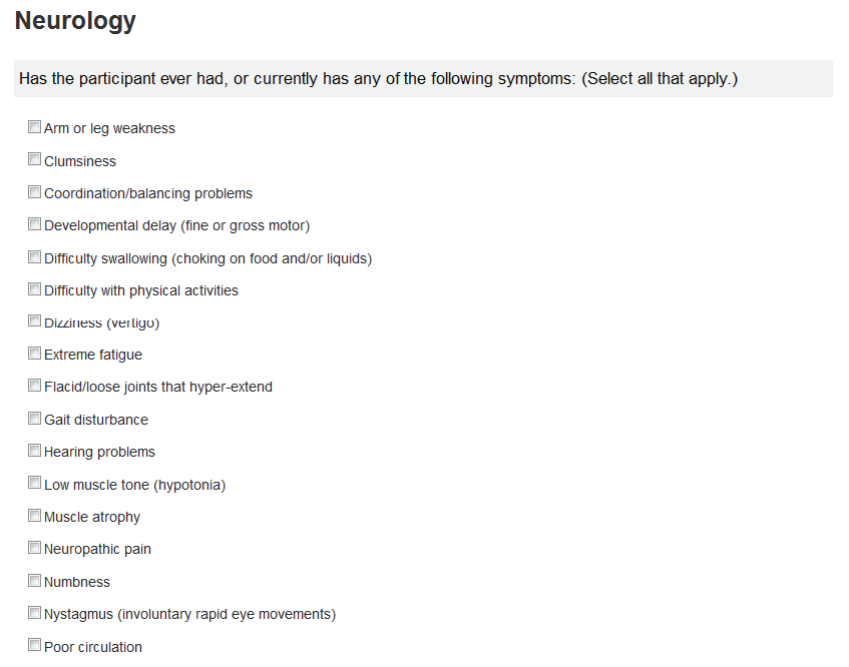
Neurology.

**Supplemental Figure 5.**
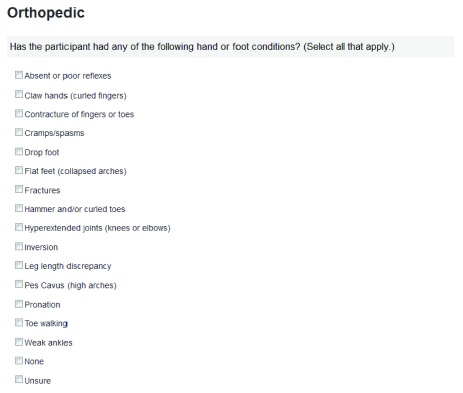
Orthopedic.

**Supplemental Figure 6.**
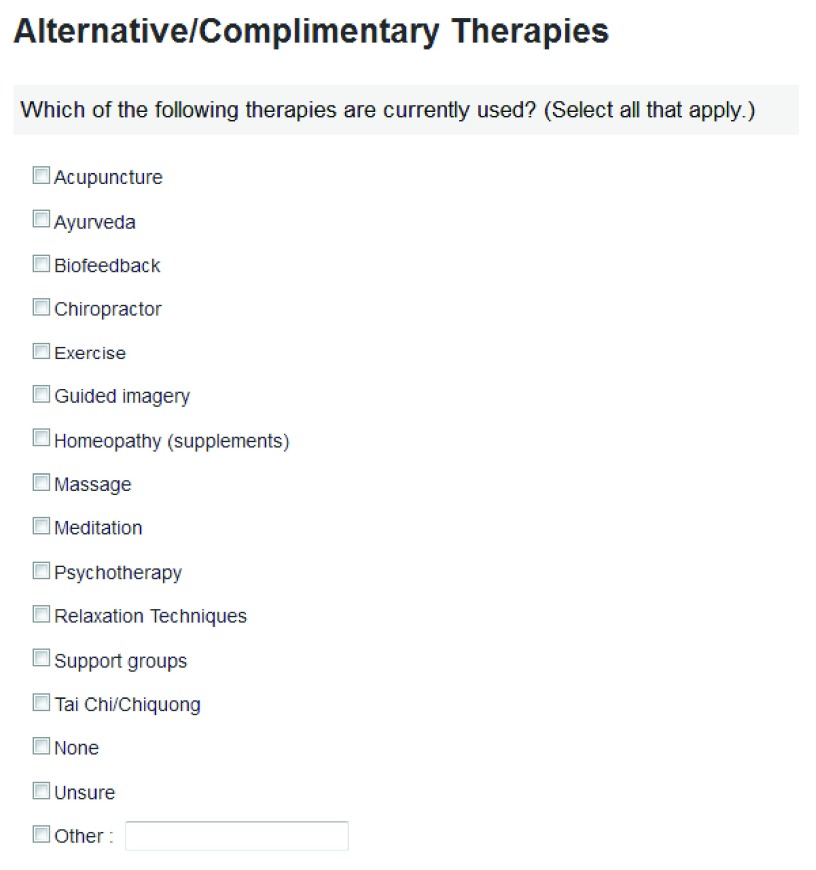
Alternative/complimentary therapies.

**Supplemental Figure 7.**
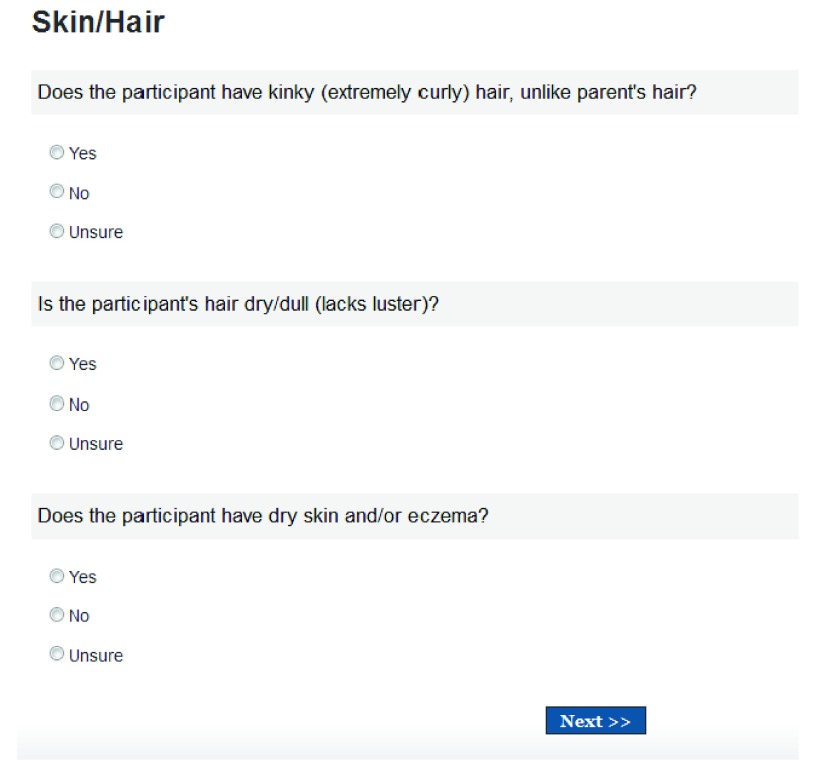
Skin/hair.

**Supplemental Figure 8.**
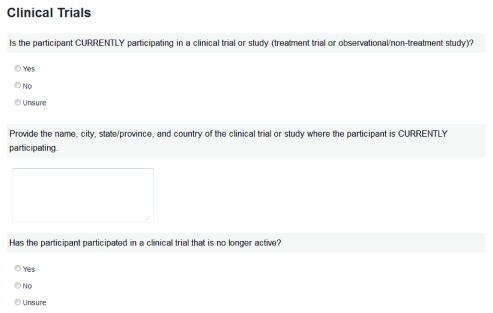
Clinical trials.

**Supplemental Figure 9.**
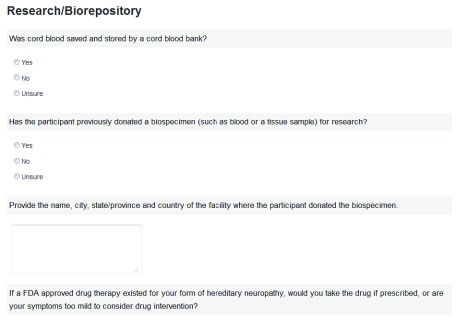
Research/biorepository.

**Supplemental Figure 11.**
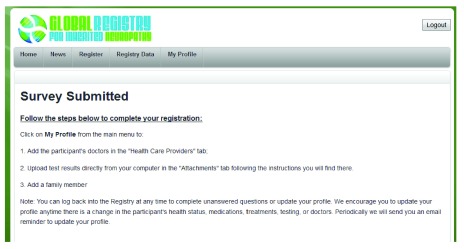
Survey submission acknowledgment.

**Supplemental Figure 12.**
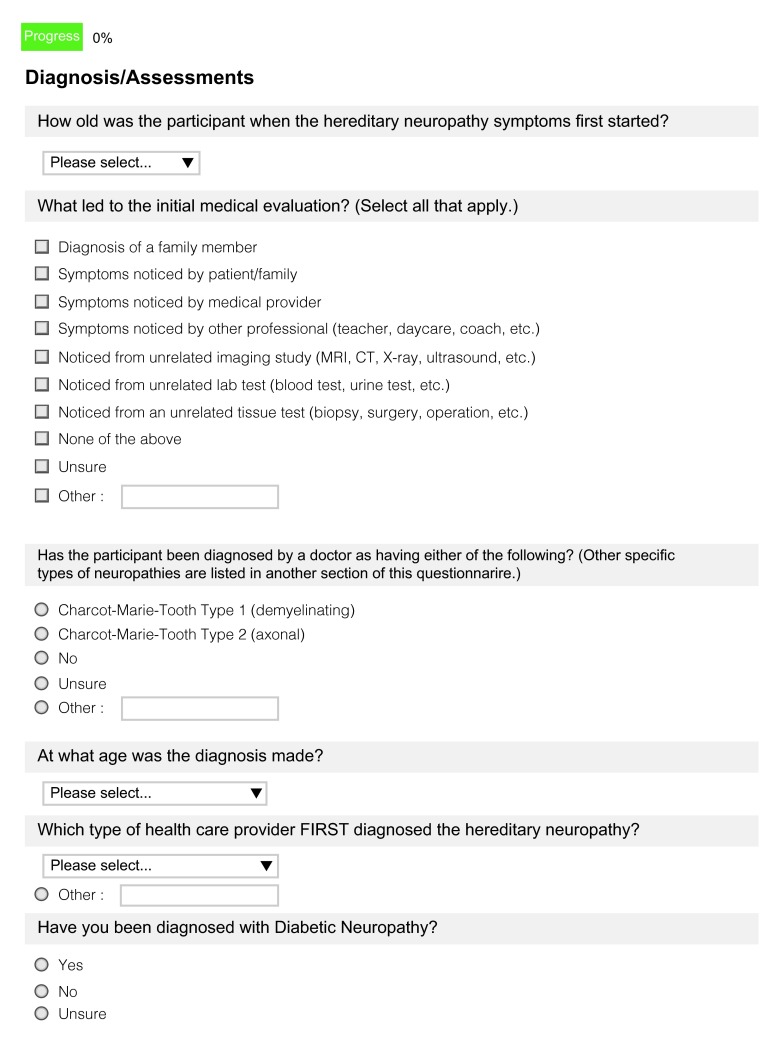
Diagnosis page in Global Registry for Inherited Neuropathy (GRIN).

## Supplementary materials

Figures relating to the Global Registry for Inherited Neuropathies.
