# Supplementary material for: Recommendations to enable drug development for inherited neuropathies: Charcot-Marie-Tooth and Giant Axonal Neuropathy
Source: F1000Res. 2014 Apr 22;3:83. Originally published 2014 Apr 3. [Version 2] doi: 10.12688/f1000research.3751.2 (PMC4023663; doi:10.12688/f1000research.3751.2)
Supplement: Peer review history

# Peer review: Referee response for version 2

**DOI:** 10.5256/f1000research.4270.r4588

**Published:** 2014-04-30

**Authors:** Jean-Michel Vallat

**Affiliations:** 1 Centre de référence neuropathies périphériques rares, CHU Limoges, Limoges, France

I have read this submission. I believe that I have an appropriate level of expertise to confirm that it is of an acceptable scientific standard.

# Peer review: Referee response for version 2

**DOI:** 10.5256/f1000research.4270.r4538

**Published:** 2014-04-23

**Authors:** Flávia L. Osório

**Affiliations:** 1 Department of Neurosciences and Behavior, University of São Paulo, São Paulo, Brazil

I approve the indexation of this revised article.

I have read this submission. I believe that I have an appropriate level of expertise to confirm that it is of an acceptable scientific standard.

# Peer review: Referee response for version 1

**DOI:** 10.5256/f1000research.4019.r4493

**Published:** 2014-04-14

**Authors:** Jean-Michel Vallat

**Affiliations:** 1 Centre de référence neuropathies périphériques rares, CHU Limoges, Limoges, France

This is a quite interesting paper which stresses that genetic treatments for neuropathies should be further investigated by neuroscientists, MD’s who have such patients in their charge, and pharmaceutical companies. On the other hand, we all agree that discoveries in molecular biology should induce increasingly personalized medicine.

Nevertheless, some points have to be explained or clarified:

- The general classification of ‘inherited neuropathies’ includes a great number of disorders. The title of this paper has to be explained in the introduction: it is clearly understandable why the authors have chosen CMT, as it is the most frequent genetic neuropathy. Now it should be discussed why they also included GAN; maybe because it is one of the rarest hereditary neuropathies? But many patients with other inherited neuropathies - such as: porphyria, Chédiak-Higashi syndrome, storage disorders with neuropathy etc. - could argue that they have been forgotten. It could be useful to present a simplified classification of inherited neuropathies in the introduction (perhaps in the form of a table?).
- The frequency of GAN is discussed and I agree is clearly underestimated: see a recent paper in Neurology ^1^ (which could be mentioned). It states: *“Giant axonal neuropathy is a rare (worldwide 50 families reported) autosomal recessive disorder”*. Actually, in our neuropathology laboratory we have about 13 GAN nerve biopsies, all from Algeria.
- I am not sure that GAN is “ *the most severe form of IN” -* even though, of course, it is very severe - leukodystrophies and storage disorders induce very severe encephalopathy.
- It should be explained that many symptoms of GAN are mostly explained by CNS involvement (impaired cognition, cerebellar dysfunction) - which is not at all the case in most CMT - so an efficient drug for this disease should also be able to cross the blood brain barrier.
- I disagree with this sentence: “ *CSF has long been exploited as a relatively non-invasive way to biopsy the CNS”*. It is true that CSF data can be very important in CNS disorders, but in no case can it be treated as a biopsy!
- Actually the preliminary results of the Pharnext phase II trial have been published. ^2^
- I agree that, at the present time, QoL should be the primary objective in therapeutic trials in CMT. I am not sure that Neuro-Qol would be a good tool as it takes into account pain and sensory signs which are not usually the main symptoms of CMT. Anyway, it will take a long time to validate such scales (PROMIS also) in CMT.
- It is correct that EIM may be a very interesting tool to follow CMT progression; so, it has to be validated - which will take some time. The possibility of utilizing this test in the coming CMT therapeutic trials could be discussed. Rutkove *et al.* ^3^ have recently shown that EIM data correlates with standard measures of ALS severity.

I have read this submission. I believe that I have an appropriate level of expertise to confirm that it is of an acceptable scientific standard.

## References

[1] IsraniAChakrabartyBGulatiSMohamedAKumarASableMNSuriV: Giant axonal neuropathy: A clinicoradiopathologic diagnosis.*Neurology.*.2014;82(9) : 83816-81710.1212/WNL.000000000000017724590247PMC3945657

[2] AttarianSDubourgOFunalotBGonnaudP-MLacourAMagyLMicallefJPéréonYPougetJStojkovicTVallatJ-MNaveK-ABertrandVCholetNGraudensEGuerassimenkoOMiletAMandelJScart GresCVialEGuedjMNabirotchkinSChumakovICohenD: A phase II randomized, placebo-controlled multicenter clinical trial of three doses of PXT3003 in 80 adult patients with CMT1A treated for 1 year. *J Peripher Nerv Syst*.2013;8(3) : 83197-19810.1111/jns5.1202523782007

[3] RutkoveSBCaressJBCartwrightMSBurnsTMWarderJDavidWSGoyalNMaragakisNJBenatarMSharmaKRNarayanaswamiPRaynorEMWatsonMLShefnerJM: Electrical impedance myography correlates with standard measures of ALS severity.*Muscle Nerve.*.2014;49(3) : 83441-44310.1002/mus.2412824273034

# Response

**Published:** 2014-04-16

**Authors:** Sean Ekins

**Affiliations:** Collaborations in Chemistry, USA

We thank the reviewer for their positive feedback. We have made most of the suggested changes. Each of the following bulletpoints refers to their comments.

 

- We do not think the title needs any explanation as it just focuses on CMT and GAN as examples of inherited neuropathies (INs). These two diseases also represent the extremes. Yes we acknowledge there are other INs but two of the authors’ families suffer from these diseases hence our interest in them specifically.
- We have added the reference for GAN as suggested.
- We have edited out *“most”* to leave *“severe”*.
- We have added *“As GAN progresses (through effects on the CNS),…”* in the text. We agree that ability to cross the BBB will be important for a GAN small molecule but we do not describe small molecule work on GAN, only gene therapy which is close to human trials. Therefore we found no appropriate place to add this in the text. We hope these comments will suffice.
- We replaced *“biopsy”* with *“sample”*.
- We have added this Pharnext reference to a meeting abstract, but the full paper is still forthcoming.
- Thank you for agreeing that QoL is important for CMT trials. As neither Neuro QoL or PROMIS have been used to our knowledge with CMT, we feel they should at least be tried.
- Thank you for agreeing that EIM may be a very interesting tool to follow CMT progression. We have added a further reference on ALS and EIM as you suggested.

# Peer review: Referee response for version 1

**DOI:** 10.5256/f1000research.4019.r4344

**Published:** 2014-04-07

**Authors:** Flávia L. Osório

**Affiliations:** 1 Department of Neurosciences and Behavior, University of São Paulo, São Paulo, Brazil

In the present article the authors review the literature on inherited neuropathies - especially Charcot-Marie-Tooth and Giant Axonal Neuropathy - based on recent clinical evidence. The authors then offer several suggestions to overcome gaps in this area of knowledge, with criticism and argumentation.

I approve this article for indexation. My only suggestion is that the authors remove figures 1 and 2 from the main body of the article and include them in the Supplementary materials instead.

I have read this submission. I believe that I have an appropriate level of expertise to confirm that it is of an acceptable scientific standard.

# Response

**Published:** 2014-04-16

**Authors:** Sean Ekins

**Affiliations:** Collaborations in Chemistry, USA

We thank the reviewer for their positive feedback. We have made the suggested changes and removed the two figures to the supplemental materials.
